# Genetic Diversity, Population Structure, and Demographic History of Anadromous Hilsa Shad (*Tenualosa ilisha*) Across Diverse Geographical Regions: A Comparative Study Between Bangladesh and Iraq

**DOI:** 10.1002/ece3.73524

**Published:** 2026-04-22

**Authors:** Md. Nurul Alam, Mohd Golam Quader Khan, Md. Bazlur Rahman Mollah, Md. Samsul Alam, Sajid Saad Hasan, Md. Shahidul Islam

**Affiliations:** ^1^ Institute of Biotechnology Bangladesh Agricultural University Mymensingh Bangladesh; ^2^ Department of Fisheries Biology and Genetics Bangladesh Agricultural University Mymensingh Bangladesh; ^3^ Department of Poultry Science Bangladesh Agricultural University Mymensingh Bangladesh; ^4^ Fisheries and Marine Resources, College of Agriculture University of Basrah Basra Iraq

**Keywords:** fisheries management, genetic diversity, mitochondrial DNA, population structure, *Tenualosa ilisha*

## Abstract

The anadromous Hilsa shad ( *Tenualosa ilisha* ) is a commercially and ecologically important fish species distributed across the Bay of Bengal, the Arabian Sea, and the river systems of South and Southeast Asia and the Middle East. Despite its significance, the genetic diversity, population structure, and demographic history of *T. ilisha* populations are poorly understood, particularly across the regions. This study provides the first comparative genetic analysis with extensive sampling of *T. ilisha* populations from Bangladesh and Iraq, utilizing mitochondrial DNA markers—Cytochrome C Oxidase subunit I (*COI*) and Cytochrome b (*CYTB*) genes—to assess genetic variation, population connectivity, and evolutionary history. A total of 279 individuals were collected from nine locations representing coastal, riverine, estuarine, mangrove forest, and floodplain environments, and 267 mitochondrial DNA sequences from the two genes (*COI* and *CYTB*) were analyzed using concatenated datasets. Genetic analyses revealed significant inter‐regional genetic differentiation, with Bangladeshi populations exhibiting higher genetic diversity, while Iraqi populations displayed genetic homogeneity. Population structure analyses using analysis of molecular variance and F‐statistics (F_ST_) demonstrated strong genetic differentiation between Bangladeshi and Iraqi populations, with inter‐regional variation accounting for over 69% of the total genetic variation. Intraregional differentiation was low among Bangladeshi populations, suggesting gene flow, whereas Iraqi populations showed no significant genetic differentiation, indicating a single, genetically uniform stock. Haplotype network analyses supported inter‐regional isolation and intraregional connectivity, with no shared haplotypes between Bangladesh and Iraq. Demographic analyses indicated recent population expansion in most Bangladeshi populations, whereas the Iraqi populations exhibited demographic stability. Expansion events in Bangladesh occurred during the late Pleistocene. Overall, the findings highlight contrasting genetic patterns between the two regions and emphasize that *T. ilisha* should not be considered a single panmictic stock. These results provide important insights for developing region‐specific conservation and management strategies for sustaining hilsa fisheries.

## Graphical abstract

To investigate genetic diversity, population structure and demographic history of the anadromous fish Hilsa shad ( *Tenualosa ilisha* ), fish collected from two populations of Iraq and seven populations of Bangladesh were subjected to the mitochondrial DNA *COI* and *CYTB* marker analysis. Population‐specific haplotypes found in Iraq and Bangladesh suggested different lineages of the species present in Bangladesh and Iraq possibly resulting from region‐specific evolution due to large geographic distances and environmental variations. No or very little genetic variation was found in fish of Iraqi populations whereas fish from different populations of Bangladesh showed substantial level of genetic variation to some extent indicating the requirements of different conservation and management strategies for different Hilsa shad regions.

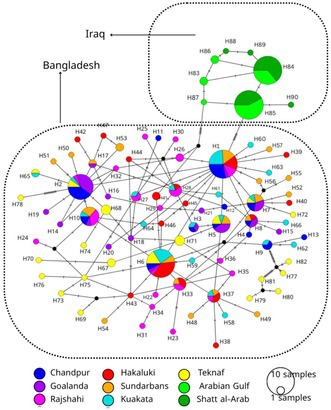

## 1 Introduction

Hilsa shad ( *Tenualosa ilisha* ) is an anadromous species with high ecological and socioeconomic importance (Alam et al. 2025). It is found in the Bay of Bengal, the Arabian Sea, and adjoining river systems across South Asia, Southeast Asia, and parts of the Middle East (Hossain et al. 2016; Bandara and Wijewardene 2023). Apart from being a major fish food for millions of people, the hilsa also plays an important role in the fishing industry of India, Bangladesh, and Myanmar. The hilsa shad is the largest single‐species fishery in Bangladesh (Alam et al. 2021). According to DoF (2024), the average production of hilsa was 5,29,487 metric tons during the fiscal year 2023–2024, contributing 10.55% of total fish production in the country. Hilsa shad in Bangladesh also has cultural significance beyond its economic contribution (Rahman 2020; Asadujjaman et al. 2022), and it is a source of livelihood for the local fishing communities. In Sindh of Pakistan, Southern Iran and Iraq, it is one of the most preferred and valuable commodities (Panhwar and Liu 2012; Vayghan and Ghanbarzadeh 2022). Hilsa previously comprised more than 90% of the total catch in Iraq's Shatt Al‐Arab River during the 1960s and 1970s and were historically dominant. It dwindled to less than 6% of the catch by the year 2020 (Mohamed 2022). Bangladesh has taken a combination of regulatory (Rahman et al. 2020) and community‐based (Asadujjaman et al. 2022) hilsa management initiatives, as well as scientific interventions (Mustafa 2020) to boost hilsa output. In any event, however, Iraq still does not possess efforts of this magnitude. Although Bangladesh's initiatives have increased hilsa production and reversed long‐standing declines in recent decades, problems still remain. The freshwater catch in Bangladesh is declining, and marine catches of hilsa are increasing. Hilsa fisheries are still threatened by overfishing and habitat degradation. This highlights the need for further research to create region‐specific, effective management procedures for the species, taking local ecological and socioeconomic conditions into consideration. Information on genetic diversity, population structure and demographic history of this species is essential for successful conservation and management measures as these factors determine how populations survive, adapt, and respond to exploitation or environmental change. Genetic diversity is an important basis for a species' adaptability and survival in changing environments (Birader 2023). Demographic history provides a foundation for understanding how *T. ilisha* has responded to past climatic and environmental changes and helps predict its future adaptability. Population structure, which reflects the distribution of genetic variation within and among populations, is shaped by factors such as geographic isolation, reproductive dynamics, and environmental heterogeneity. Evaluating these parameters is therefore crucial for developing effective conservation and management strategies.

Although several studies on the population genetics of *T. ilisha* are available in Bangladesh and India (Rahman and Nævdal 2000; Shifat et al. 2003; Ahmed et al. 2004; Lal et al. 2004; Brahmane et al. 2006; Mazumder and Alam 2009; Brahmane et al. 2013; Asaduzzaman, Wahab, et al. 2019; Asaduzzaman, Igarashi, et al. 2019; Mohindra et al. 2019; Choudhury and Das 2021; Sarker et al. 2021; Khatun et al. 2022), little is known about the studies on this species from the Iraqi region. Additionally, no studies have compared the genetic diversity, population structure, and demographic history of these *T. ilisha* populations from Bangladesh and Iraq. Population structuring in *T. ilisha* has been previously studied between the Bay of Bengal and the Arabian Sea (Behera et al. 2015; Abdullah et al. 2022; Habib et al. 2022), however, a more thorough assessment is required. Although different markers were used in previous studies, small sample sizes, limited sampling sites, and low‐resolution methods have led to an incomplete insight into the population structure and demographic history of *T. ilisha* in Bangladesh and Iraq. Rahman and Nævdal (2000) and Lal et al. (2004) analyzed allozyme markers to study the population genetics of *T. ilisha* in Bangladesh waters. However, these markers have constraints when compared with DNA‐based markers (e.g., low polymorphism, limited genomic coverage, susceptibility to selective pressures) that may make them potential for biased interpretations (Liu and Furnier 1993; Silva and Skibinski 2008; Neetu and Chaudhry 2011). Ahmed et al. (2004) studied the hilsa population structure by RFLP (restriction fragment length polymorphism) analysis of the mtDNA D‐loop region. But that study was limited to sampling just seven fish from each of three sites in Bangladesh. Studies by Brahmane et al. (2006) and Shifat et al. (2003) were preliminary with small sample sizes. They employed the RAPD (randomly amplified polymorphic DNA) fingerprinting method. RAPD markers are unsuitable for detailed study of population structure because of their low resolution and poor reproducibility compared with sequencing‐based markers. Mazumder and Alam (2009) studied population structure and diversity in hilsa by using RFLP analysis of mtDNA D‐loop region. However, their study was limited to five sampling locations and smaller (18 fish per location) sample sizes than are needed for reliable conclusions. They ultimately suggested sequencing‐based mtDNA analysis with extensive sampling for more accurate results. Asaduzzaman, Wahab, et al. (2019) and Asaduzzaman, Igarashi, et al. (2019) investigated the population structure of hilsa in Bangladesh waters based on SNP (single‐nucleotide polymorphism) markers, but their investigation did not include all important habitat types such as floodplains and mangrove forests. Although Sarker et al. (2021) included more sampling sites across Bangladesh in their genetic analysis of hilsa populations, the sample sizes per location were relatively small (1–10 individuals). The above constraints from the previous studies warrant conducting more extensive studies with larger sample sizes, wide geographical coverage and detailed resolution approaches to unravel genetic parameters of *T. ilisha* populations. We conducted our research to investigate genetic diversity, population genetic structure and demographic history using concatenated sequences for two mitochondrial genes (*COI* and *CYTB*) in *T. ilisha* populations from a wide range of habitats across Bangladesh and Iraq. The goal of this research was to validate the findings observed in other studies, and also to compare the population genetic parameters of Bangladesh and Iraqi populations. Among numerous markers, mitochondrial DNA has been widely utilized to infer genetic diversity, population structure, and demographic history in fish species due to their high mutation rates, maternal inheritance, and lack of recombination (Matiiuk 2022; Nehemia and Mwakalesi 2024; Mandal et al. 2025). The concatenated sequences for *COI* and *CYTB* in this study provide information on mitochondrial genetic diversity and population structure of *T. ilisha* within and across regions. This research is the first‐ever direct comparison of *T. ilisha* populations from these two regions. Therefore, comparing the genetic parameters of populations from Bangladesh and Iraq can provide a rare opportunity to understand how geography, habitat conditions, and human activities contribute to shaping the gene pool of this species. The information from this study will assist in developing region‐specific conservation and management strategies to ensure sustainable production of the Hilsa shad.

## 2 Methodology

### 2.1 Sample Collection

**FIGURE 1:**
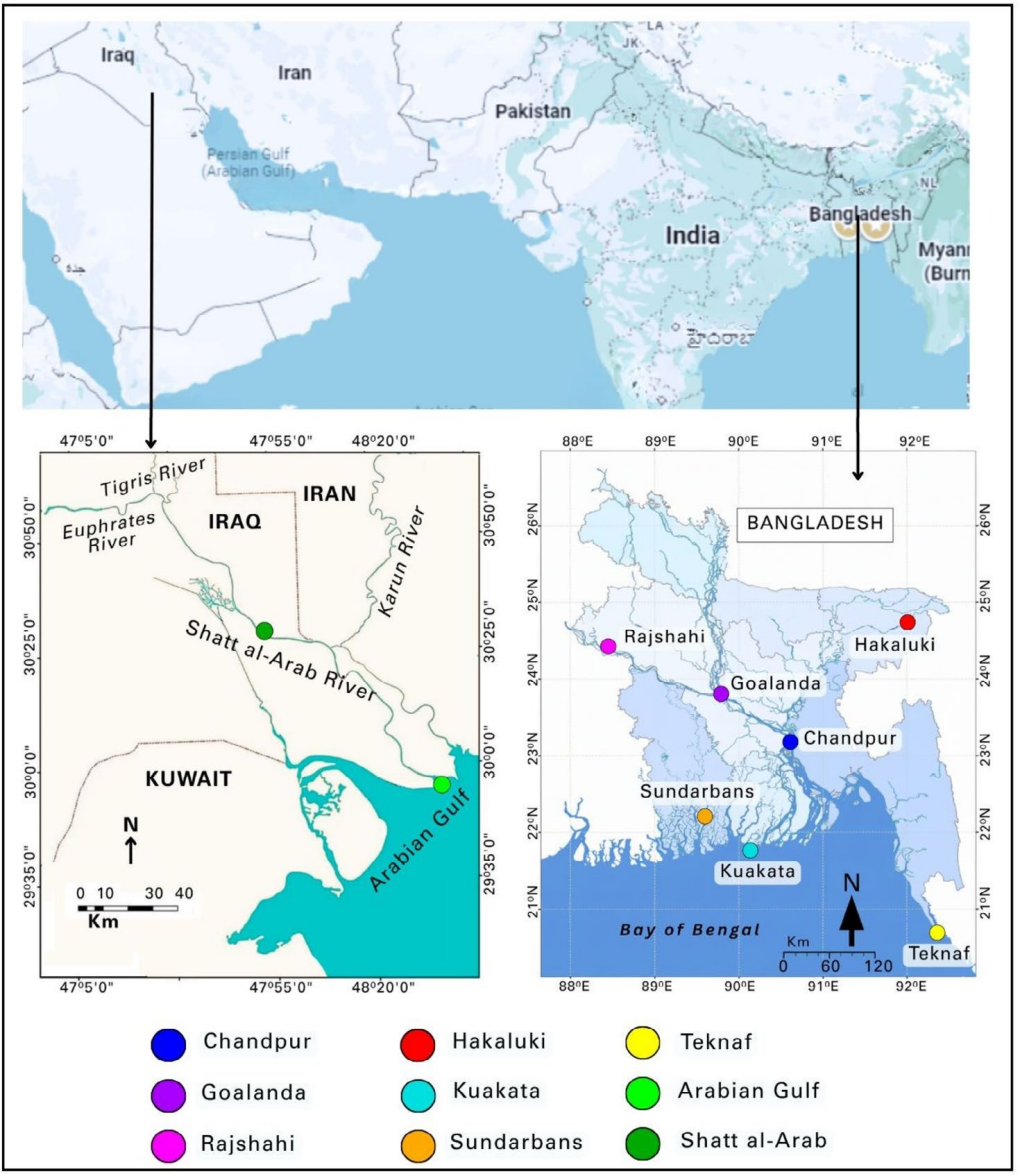
Sampling sites of *T. ilisha* across Bangladesh and Iraq. Colored spots on the map indicate the locations where *T. ilisha* samples were collected: In Bangladesh (Chandpur, Goalanda, Rajshahi, Hakaluki, Kuakata, Sundarbans, and Teknaf) and in Iraq (the Arabian Gulf and Shatt al‐Arab).

Samples were collected according to the Protection and Conservation of Fish Rules, 1985 of the Government of the People's Republic of Bangladesh and the Law Regulating the Exploitation and Protection of Aquatic Life (Law No. 48 of 1976) of the Republic of Iraq. Fish from different locations were collected using fishing boats from October 2020 to September 2023. Pectoral fin tissues from 279 fresh individuals of nine locations were preserved in 95% ethanol3. Ethanol‐preserved fin tissues were kept in a cooler box during transportation and stored at −20°C in the laboratory until DNA extraction. The sampling sites included seven locations in Bangladesh, namely Chandpur, Goalanda, Rajshahi, Hakaluki, Kuakata, Sundarbans, and Teknaf, and two locations in Iraq, namely, the Arabian Gulf and the Shatt al‐Arab (Table 1 and Figure 1). Sampling sites were selected to represent diverse aquatic habitats, including coastal marine zones, downstream riverine areas, midstream and upstream freshwater bodies, and seasonal water bodies (Table 1).

**TABLE 1:** Details of *T. ilisha* sample collection across Bangladesh and Iraq.

Sl. No.	Locations	Sources	Habitat type	GPS location	No. of samples	Date of collection
1	Chandpur	Meghna River, Chandpur district	Midstream river	23° 13′09.0″ N 90° 37′08.0″ E	30	8/10/2020
2	Goalanda	Padma River, Goalandaghat, Rajbari district	Midstream river	23° 46′11.3″ N 89° 47′07.4″ E	31	14/01/2021
3	Rajshahi	Padma upstream, Godagari, Rajshahi district	Upstream river	24° 26′22.6″ N 88° 19′19.7″ E	31	16/11/2021
4	Hakaluki	Hakaluki haor, upstream of the Meghna River, Moulvibazar district	Floodplain ecosystem	24° 41′42.7″ N 92° 00′52.5″ E	32	17/09/2021
5	Sundarbans	Pasur River, Sundarbans, Khulna district	Mangrove ecosystem, Coastal habitat	22° 28′39.0″ N 89° 35′18.0″ E	31	04/11/2021
6	Kuakata	Bay of Bengal, Kuakata, Patuakhali district	Marine	21° 48′50.3″ N 90° 07′19.6″ E	30	27/09/2021
7.	Teknaf	Naf River, Teknaf, Cox's Bazar district	Estuarine	20° 51′42.1″ N 92° 18′52.2″ E	32	30/9/2023
8.	Arabian Gulf	Arabian Gulf, Iraq	Marine	29° 54′30.4″ N 48° 38′08.8″ E	31	20/07/2022
9.	Shatt al‐Arab	Shatt al‐Arab, Iraq	Midstream river	30° 28′45.7″ N 47° 53′30.6″ E	31	20/07/2022

### 2.2 Extraction, Confirmation, and Quantification of Total Genomic DNA

Total genomic DNA was extracted from alcohol‐preserved pectoral fin tissues of *T. ilisha* using the phenol, chloroform, isoamyl alcohol extraction, and ethanol precipitation method described by Islam and Alam (2004) with some modifications. In brief, approximately 25 mg of fin tissue was finely chopped, homogenized, and digested with proteinase‐K in a lysis buffer[100 mM Tris–HCl, pH 8.0, 10 mM ethylenediaminetetraacetic acid (EDTA), 250 mM NaCl and 1% sodium dodecyl sulfate (SDS)] overnight at 37°C. DNA was purified by successive extractions: first using phenol: chloroform: isoamyl alcohol (25:24:1, v/v/v), and then with chloroform: isoamyl alcohol (24:1, v/v) to remove residual phenol and proteins. DNA was precipitated by adding 0.6 volumes of isopropanol, followed by centrifugation at 13,000 rpm for 15 min. The resulting pellet was resuspended in TE buffer (100 mM Tris–HCl, 1 mM EDTA, pH 8.0). DNA was re‐precipitated by adding 2.5 volumes of ice‐cold absolute ethanol in the presence of 0.1 volume of 3 M sodium acetate (pH 5.2) and pelleted by centrifugation. The pellets were washed with 70% ethanol, air‐dried at room temperature, and resuspended in 60 *μ*L of TE (10 mM Tris‐base and 1 mM EDTA) buffer. All glassware, microcentrifuge tubes, micropipette tips, tissue homogenizers, forceps, scissors, TE buffer, and lysis buffer were thoroughly autoclaved and maintained under sterile conditions. DNA quality was assessed by 0.8% agarose gel electrophoresis, and concentration was determined using a NanoDrop One spectrophotometer (Thermo Fisher Scientific, USA).

### 2.3 PCR Amplification of Target Region of mtDNA COI and CYTB

A 953 bp fragment of *COI* and an 802 bp fragment of *CYTB* were amplified from the total genomic DNA of *T. ilisha* specimens using a thermal cycler (Biometra TOne, Germany). The primers used were as follows: for *COI*, the forward primer 5′‐TCAACCAACCACAAAGACATTGGCAC‐3′ (Ward et al. 2005) and the reverse primer 5′‐GTGGCCAGTCAGCTAAAGACTTT‐3′ (designed in this study); for *CYTB*, the forward primer 5′‐GACTTGAAGAACCACCGTTG‐3′ (Near et al. 2000) and the reverse primer 5′‐TGGGTCTCCTAGTAGGTTTG‐3′ (designed in this study). PCR amplification was carried out in a 30 *μ*L reaction volume containing 6 *μ*L (conc. 50 ng/μL) of template DNA, 15 *μ*L of 2× Master Mix (New England Biolabs, UK), 0.3 *μ*L of each primer at 10 *μ*M (Macrogen, South Korea), and 8.4 *μ*L of sterile deionized water. The thermal cycling profile for *COI* amplification included an initial denaturation at 95°C for 5 min; 35 cycles of denaturation at 94°C for 1 min, annealing at 47°C for 1.5 min, and extension at 72°C for 2 min; followed by a final extension at 72°C for 7 min. *CYTB* amplification followed the same profile, except for the annealing temperature, which was set to 60°C for 1.5 min. Successful amplifications of both genes were confirmed by 2% agarose gel electrophoresis.

### 2.4 Purification and Sequencing of PCR Products

PCR products were purified using the QIAquick Gel Extraction Kit (Qiagen), following the manufacturer's instructions, and quantified using a NanoDrop One spectrophotometer (Thermo Fisher Scientific, USA). Purified PCR products were Sanger‐sequenced using the 3500 Genetic Analyzer (Applied Biosystems) at the National Institute of Biotechnology, Savar, Dhaka, Bangladesh, and the 3730xL Genetic Analyzer (Applied Biosystems) at Wuhan Jinkairui Biological Co. Ltd., China.

### 2.5 Sequence Data Analysis

Chromatograms (visual representations of the fluorescent signals detected during DNA sequencing) were visually inspected for anomalies such as sequencing artifacts, ambiguous base calls, or signal noise, using Chromas version 2.6.6 (Technelysium Pty Ltd). Based on sequence quality, 267 out of 279 sample sequences were selected for the analysis of both *COI* and *CYTB*. Multiple sequence alignment for downstream analyses was performed using the MUSCLE algorithm with default settings in MEGA version 11.0.13 (Tamura et al. 2021). Sequences were trimmed to 813 bp and 726 bp for *COI* and *CYTB*, respectively, by removing low‐quality flanking regions. Nucleotide sequences were subjected to NCBI BLAST to confirm species identity. *COI* and *CYTB* sequences were concatenated to make 1539 bp. Aligned sequences were collapsed into haplotypes by removing invariable sites and merging identical sequences using DnaSP v6.12.03 (Rozas et al. 2017). Genetic diversity indices—including haplotype number, haplotype diversity, nucleotide diversity, and the number of segregating sites—were calculated using DnaSP v6.12.03 with default settings. Population structure was assessed using AMOVA and F‐statistics in Arlequin v3.5 (Excoffier and Lischer 2010). Tests of statistical significance for AMOVA and F‐statistics were conducted with 10,000 permutations. TCS (a statistical parsimony algorithm used to construct haplotype network developed by Templeton et al. 1992) haplotype network analysis was performed using PopArt version 1.7 (Clement et al. 2002; Leigh and Bryant 2015), based on a haplotype Nexus file generated in DnaSP v6.12.03. Demographic history was inferred using neutrality tests (Tajima's *D* and Fu's *Fs*) with 1000 simulated samples and mismatch distribution with 10,000 bootstrap replicates under sudden expansion model conducted in Arlequin v3.5. Harpending's raggedness index (RI) was used to evaluate the smoothness of mismatch distribution (Harpending 1994). The sum of squared deviations (SSD) was calculated to assess whether the observed mismatch distribution significantly deviates from the expected distribution under a population expansion model.

The time since population expansion was estimated from the mismatch distribution parameter (τ) following the sudden expansion model implemented in Arlequin. The relationship τ=2ut was used, where t represents the time since expansion in generations and u is the mutation rate per sequence per generation. The parameter u was calculated as u=μ×k, where μ is the mutation rate per site per generation and k is the length of the DNA sequence analyzed. The time since expansion in years was then obtained by multiplying the estimated number of generations by the assumed generation time. A mutation rate of 1%–3% per million years (Wang et al. 2012) and a generation time of 1.2 years (Ahmed et al. 2020) were applied for calculation.

## 3 Results

### 3.1 Genetic Diversity

Genetic diversity, including the number of segregating sites, number of haplotypes, haplotype diversity, and nucleotide diversity, is summarized in Table 2. Seventy‐nine segregating sites and 90 haplotypes were observed in all sequences. Haplotype diversity among Bangladeshi populations ranged from 0.878 to 0.968; in Iraqi populations, it ranged from 0.598 to 0.648 (Table 2). Among all sampling sites, the Bangladeshi population Teknaf exhibited the highest haplotype diversity (0.968), while Iraqi population Shatt al‐Arab showed the lowest (0.598). Nucleotide diversity ranged from 0.00166 to 0.00706 among Bangladeshi sites, and from 0.00049 to 0.00053 in Iraqi sites (Table 2). Notably, the Teknaf population exhibited the highest nucleotide diversity, while the Shatt al‐Arab population showed the lowest among all populations.

**TABLE 2:** Genetic diversity of *T. ilisha* populations sampled from Bangladesh and Iraq based on concatenated mtDNA partial *COI* and *CYTB* sequences.

Populations	Number of sequences	Number of segregating sites	Number of haplotypes	Haplotype diversity	Nucleotide diversity
Chandpur	30	12	13	0.878	0.00166
Goalanda	29	14	16	0.906	0.00168
Rajshahi	30	15	20	0.966	0.00218
Hakaluki	30	15	16	0.897	0.00168
Sundarbans	28	20	19	0.966	0.00229
Kuakata	30	18	20	0.931	0.00189
Teknaf	31	42	21	0.968	0.00706
Arabian Gulf	29	2	5	0.648	0.00053
Shatt al‐Arab	30	4	5	0.598	0.00049
Overall	267	79	90	0.955	0.00425

### 3.2 Population Structure

The AMOVA results for *T. ilisha* across different populations are presented in Table 3. AMOVA across all populations revealed that 69.42% of the genetic variation (*p* = 0.02881 ± 0.00152) was attributable to differences among groups (regions), while 2.60% (*p* = 0.000) was due to variation among populations within regions, and 27.98% (*p* = 0.000) was due to variation within populations. AMOVA results for the Bangladeshi populations showed a markedly different pattern. Only 7.90% of the variation occurred among populations (*p* = 0.0000), while 92.10% was within populations. AMOVA analysis of Iraqi populations did not reveal any statistically significant variation among populations (*p* = 0.16604 ± 0.00357); the variation was primarily within populations (97.84%).

**TABLE 3:** AMOVA results for nine populations of *T. ilisha* based on concatenated mtDNA partial *COI* and *CYTB* sequences.

	Source of variation	df	Sum of squares	Variance components	Percentage of variation	*p*
*Nine populations*	Among groups(regions)	1	390.390	4.17824 Va	69.42	0.02881 ± 0.00152
	Among populations within groups(regions)	7	44.271	0.15636 Vb	2.60	0.000
	Within populations	258	434.432	1.68385 Vc	27.98	0.000
	Total	266	869.094	6.01844		
*Bangladeshi populations*	Among populations	6	43.619	0.17571 Va	7.90	0.000
	Within Populations	201	411.915	2.04933 Vb	92.10	
	Total	207	455.534	2.22504		
*Iraqi populations*	Among populations	1	0.652	0.00872 Va	2.16	0.16604 ± 0.00357
	Within populations	57	22.517	0.39504 Vb	97.84	
	Total	58	23.169	0.40376		

Pairwise genetic differentiations (F‐statistics) along with corresponding *p*‐values are presented in Table 4. Strong genetic differentiation was observed between the Bangladeshi and Iraqi populations, with statistically significant F_ST_ values ranging from 0.570 to 0.845. In case of within‐region populations, no significant F_ST_ values (*p* = 0.162 ± 0.004) were detected between the two Iraqi populations whereas statistically significant but lower levels of F_ST_ values ranging from 0.044 to 0.208 were found between some Bangladeshi populations (Table 4).

**TABLE 4:** Pairwise genetic differentiation (F_ST_) between *T. ilisha* populations based on concatenated mtDNA partial *COI* and *CYTB* sequences (below diagonal) and corresponding *p*‐ values (upper diagonal).

Locations	Chandpur	Goalanda	Rajshahi	Hakaluki	Sundarbans	Kuakata	Teknaf	Arabian Gulf	Shatt al‐Arab
Chandpur	—	0.1881 ± 0.0035	0.0044 ± 0.0006	0.0010 ± 0.0003	0.5116 ± 0.0052	0.112 ± 0.0032	0.0008 ± 0.0003	0.000 ± 0.000	0.000 ± 0.000
Goalanda	0.014 ns	—	0.000 ± 0.000	0.000 ± 0.000	0.0182 ± 0.0014	0.0002 ± 0.0001	0.0000 ± 0.0000	0.000 ± 0.000	0.000 ± 0.000
Rajshahi	0.077**	0.129***	—	0.32561 ± 0.0046	0.0775 ± 0.0028	0.1752 ± 0.0037	0.0001 ± 0.0001	0.000 ± 0.000	0.000 ± 0.000
Hakaluki	0.121**	0.208***	0.003 ns	—	0.0368 ± 0.0018	0.3263 ± 0.0043	0.0001 ± 0.0001	0.000 ± 0.000	0.000 ± 0.000
Sundarban	−0.005 ns	0.046*	0.027 ns	0.044*	—	0.6534 ± 0.0051	0.0043 ± 0.0006	0.000 ± 0.000	0.000 ± 0.000
Kuakata	0.024 ns	0.099***	0.013 ns	0.003 ns	−0.010 ns	—	0.0003 ± 0.0002	0.000 ± 0.000	0.000 + −0.000
Teknaf	0.095***	0.108***	0.103***	0.124***	0.080**	0.097***	—	0.000 ± 0.000	0.000 ± 0.000
Arabian Gulf	0.833***	0.833***	0.807***	0.839***	0.800***	0.821***	0.570***	—	0.162 ± 0.004
Shatt al‐Arab	0.839***	0.839***	0.814***	0.845***	0.807***	0.827***	0.578***	0.022 ns	—

*Note:* * = *p* < 0.05, ** = *p* < 0.01, *** = *p* < 0.001.

Among the Bangladeshi populations showing significant levels of F_ST_, the highest value (0.208; *p* = 0.000 ± 0.000) was observed between the Goalanda and Hakaluki populations, whereas the lowest value (0.044; *p* = 0.0368 ± 0.0018) was found between the Sundarbans and Hakaluki. Specimens of *T. ilisha* from Teknaf were markedly different from those of other Bangladeshi locations, showing significant differentiation with all other Bangladeshi populations, with F_ST_ values ranging from 0.080 to 0.124. Fish from Rajshahi were significantly different from those in Goalanda and Chandpur populations. However, no significant differentiation was observed in the Chandpur versus Goalanda, Sundarbans versus Kuakata, Rajshahi versus Hakaluki, Sundarbans versus Kuakata, Kuakata versus Hakaluki and Kuakata versus Sundarbans population pairs (Table 4). *T. ilisha* from Kuakata also did not differ from Hakaluki and Sundarbans.

### 3.3 Haplotype Network Analysis

**FIGURE 2:**
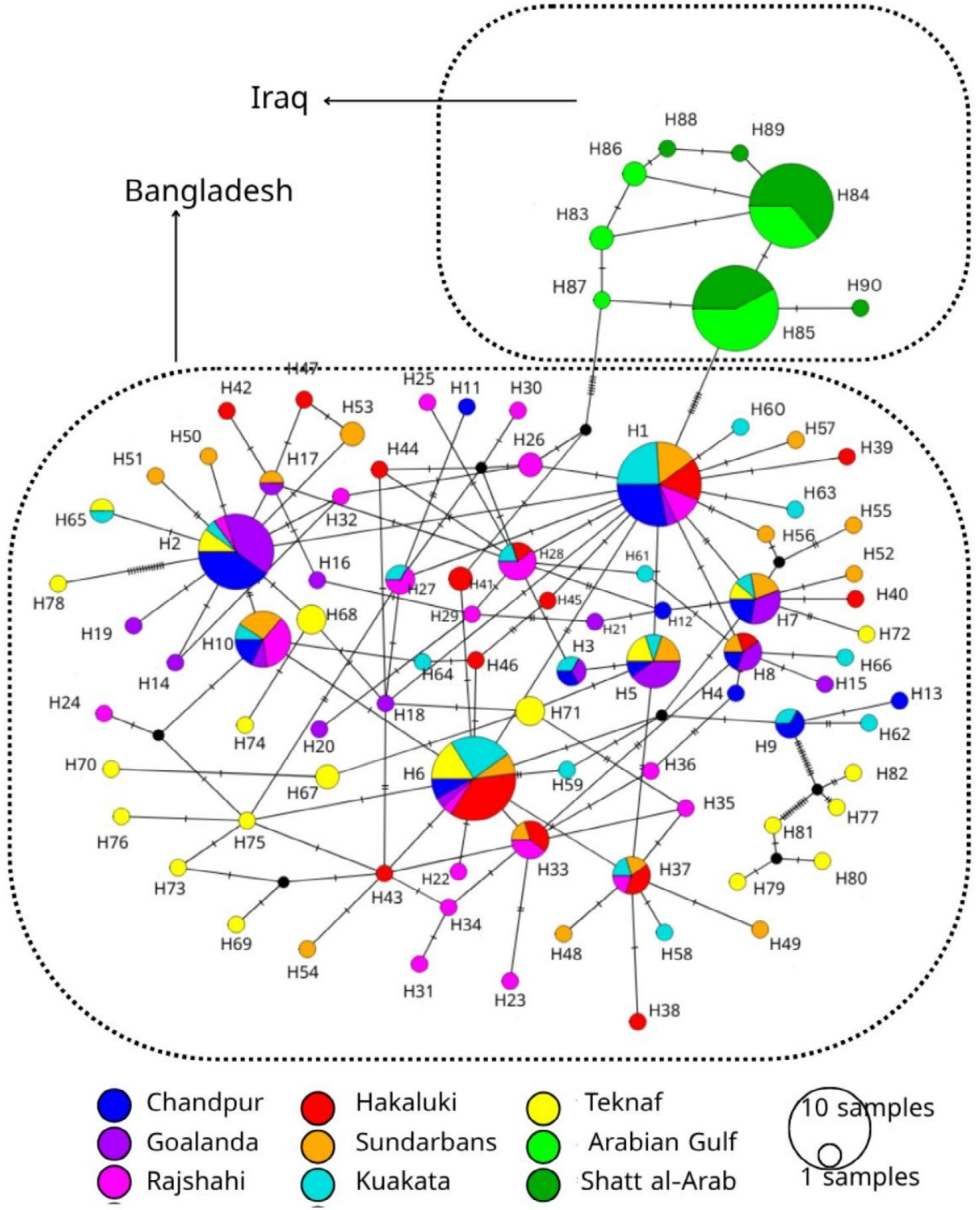
TCS haplotype network constructed from concatenated *COI* and *CYTB* sequences showing genetic relationships among *T. ilisha* populations from Bangladesh and Iraq. Each circle represents a haplotype, with the size of the circle proportional to the frequency of individuals possessing that haplotype. Colors indicate geographic origins of haplotypes, and black lines on the branches are used to indicate the number of mutational changes between two different haplotypes. H_ indicates haplotypes.

The haplotype network based on 90 haplotypes from 267 concatenated sequences indicated that no haplotypes were shared between the Bangladeshi and Iraqi populations. Out of 90 haplotypes, 82 were found in different Bangladeshi hilsa populations, and eight were found in two Iraqi populations. Within Bangladesh, haplotype 6 was common across populations and haplotype 1 was shared by all populations except the Teknaf, while in Iraq, haplotypes 84 and 85 were shared by the two populations (Figure 2). Most of the haplotypes were found population‐specific or distinct (Table S1 and Figure 2). In Bangladesh, the highest number of distinct haplotypes (16 out of 21) was found in the Teknaf population while the least number (4 out of 13) of distinct haplotypes was found in the Chandpur population. Each of the two populations of Iraq on the other hand, had three distinct haplotypes. Haplotypes of two Iraqi and most of the Bangladeshi populations were separated from one another by only one or two mutational steps. However, some Teknaf haplotypes were separated from other Bangladeshi haplotypes by more than 10 mutational steps indicating substantial genetic divergence.

### 3.4 Demographic History

**FIGURE 3:**
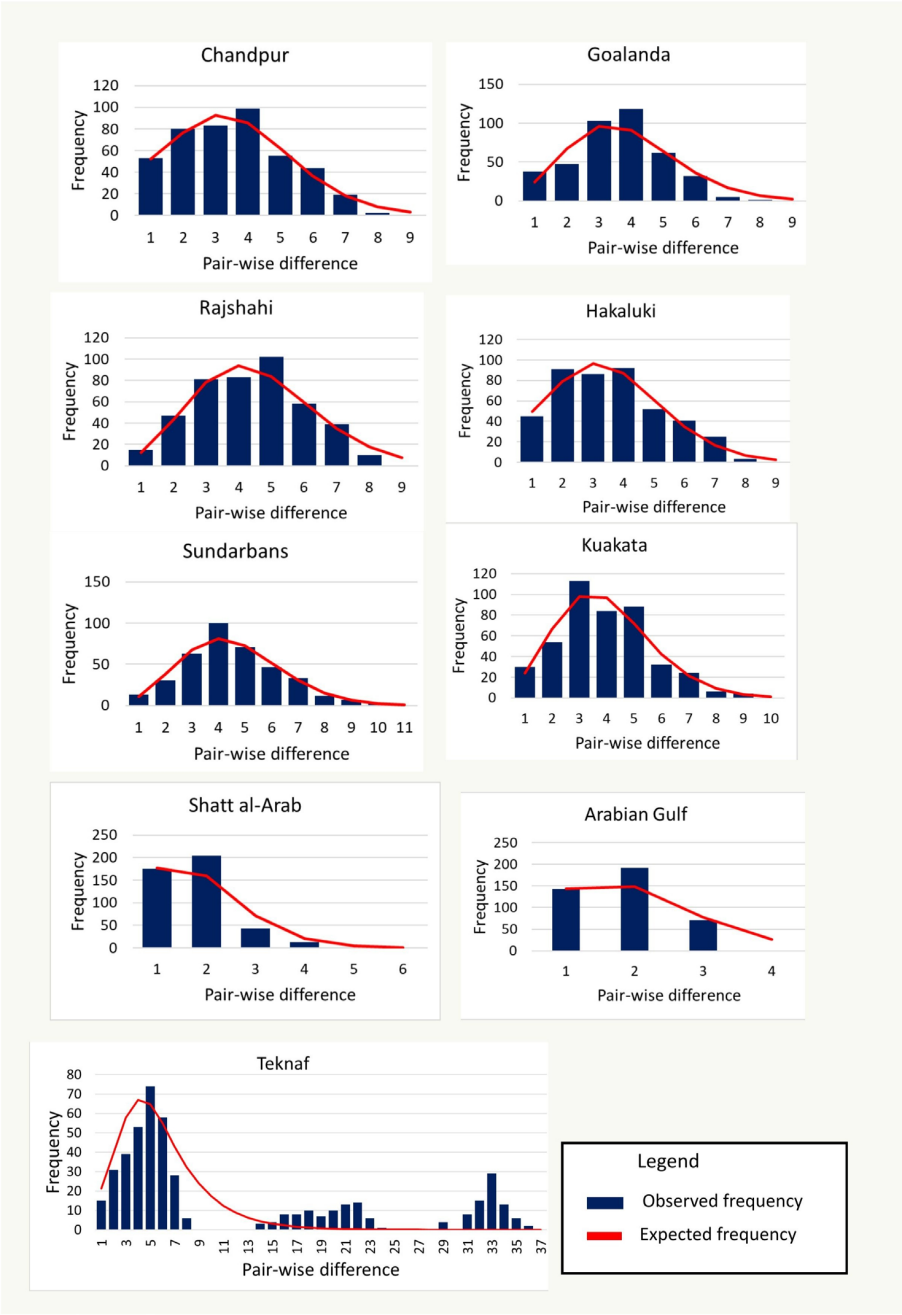
Mismatch distribution of pairwise nucleotide differences among individuals of *T. ilisha* from nine sampling locations based on concatenated mitochondrial DNA sequences (*COI* and *CYTB*). Histograms represent the observed frequency of pairwise differences, while the red curves indicate the expected frequency under a sudden population expansion model as implemented in Arlequin. Pairwise differences are shown on the x‐axis, and frequency is shown on the y‐axis.

The results of neutrality tests and mismatch distribution are presented in Table 5. None of the Tajima's D values from the nine populations were statistically significant. In Fu's Fs test, significant negative values were detected in all Bangladeshi populations except the Teknaf. In the mismatch distribution, all Bangladeshi populations showed unimodal distribution except the Teknaf (Figure 3). Iraqi populations showed nonsignificant Fu's Fs values and unimodal mismatch distributions. The sum of squared deviation and Harpending's RI was not significant in any of the nine populations. Based on the mismatch distribution parameter (τ), the estimated time since population expansion ranged from 36,500 to 138,800 years in six Bangladeshi populations that showed significant negative values for Fu's Fs test.

**TABLE 5:** Results of neutrality tests (*Tajima's D* and *Fu's Fs*) and mismatch distribution analyses for *T. ilisha* populations based on concatenated mtDNA partial *COI* and *CYTB* sequences. *p*‐values are shown in parentheses.

Populations	Neutrality tests		Mismatch distribution	
	Tajima's D	Fu's Fs	Sum of squared deviation (*p*‐value)	Harpending's Raggedness index (*p*‐value)
Chandpur	−0.51171 ns (0.35200)	−5.23147** (0.005)	0.00224 (0.82930)	0.02098 (0.90220)
Goalanda	−0.91801 ns (0.19000)	−9.75659*** (0.00000)	0.00997 (0.13920)	0.04989 (0.25250)
Rajshahi	−0.38250 ns (0.39500)	−14.06736*** (0.00000)	0.00302 (0.43600)	0.03056 (0.45630)
Hakaluki	−1.07167 ns (0.14700)	−9.49951*** (0.00000)	0.00263 (0.75600)	0.02456 (0.84040)
Sundarban	−1.11207 ns (0.11600)	−12.52395*** (0.00000)	0.00353 (0.44320)	0.03398 (0.35980)
Kuakata	−1.24505 ns (0.10800)	−15.85627*** (0.00000)	0.00518 (0.31760)	0.04470 (0.29240)
Teknaf	0.12125 ns (0.61700)	−4.11578 ns (0.07700)	0.02229 (0.1655)	0.01594 (0.69400)
Arabian Gulf	1.27355 ns (0.90100)	−1.17958 ns (0.19800)	0.01128 (0.20220)	0.13397 (0.15850)
Shatt al‐Arab	−0.63844 ns (0.31000)	−1.35749 ns (0.12400)	0.01514 (0.16230)	0.14715 (0.12020)

*Note:* * = *p* < 0.05, ** = *p* < 0.01, *** = *p* < 0.001, ns = not significant.

## 4 Discussion

This study offers substantial insights into the genetic diversity, population structure, and demographic history of anadromous Hilsa shad ( *T. ilisha* ) across nine populations from Bangladesh and Iraq. The findings indicate pronounced inter‐regional genetic differentiation and restricted gene flow, intraregional genetic connectivity, and varying degrees of genetic diversity. Notably, Bangladeshi populations displayed higher genetic diversity and more complex demographic patterns compared with those from Iraq. Consistent with our results, Habib et al. (2022) also reported high levels of genetic variation and divergence between *T. ilisha* populations from the Bay of Bengal and the Arabian Sea.

The results demonstrated differences in genetic diversity between the Bangladeshi and Iraqi hilsa populations, with Bangladeshi populations generally displaying higher levels of both haplotype and nucleotide diversity. Fish from Bangladeshi populations showed a wider and elevated range in haplotype diversity from 0.878 to 0.968, compared with the Iraqi populations, which have a narrower range (0.598–0.648). The low genetic diversity in Iraq suggests historical bottlenecks, possibly due to habitat fragmentation, overfishing, or pollution‐induced habitat degradation (Pinsky and Palumbi 2013; Klütsch et al. 2019; Sharma 2020). Conversely, the higher diversity in Bangladeshi populations may reflect larger effective population sizes, continuous gene flow, and favorable ecological conditions (Chung and Chung 2014; Schou et al. 2017; Gompert et al. 2021).

Apart from inter‐regional genetic diversity differences, notable intraregional diversity variation was also evident among populations from different ecological habitats in Bangladesh. The Teknaf population, for example, showed the highest haplotype diversity (0.968) and nucleotide diversity (0.00706) (Table 2), indicating a genetically diverse population. This high diversity could be due to the convergence of riverine and marine influences in the Teknaf population. High haplotype diversity in such estuarine populations is often associated with complex environmental conditions that promote greater genetic variability (McDonald and Yeaman 2018). In contrast, Chandpur, a Bangladeshi riverine site, showed the lowest haplotype diversity (0.878) and nucleotide diversity (0.00166) among Bangladeshi populations (Table 2). Riverine populations often display lower genetic diversity due to environmental constraints, river fragmentation and limited connectivity with other populations (Gouthier et al. 2023). But in the Padma upstream location, the Rajshahi population showed higher haplotype and nucleotide diversity (0.966, 0.00218) than the marine population Kuakata (0.931, 0.00189). Further study is needed to explore how the Rajshahi population maintains its gene diversity, or if there is a potamodromous type of *T. ilisha* population there. According to Hossain et al. (2016), a small subset of the Hilsa shad population completes its life cycle in rivers. The upstream and downstream connectivity of the Padma River is significantly compromised due to low water inflow during the dry season. This disconnection is driven by the decreased flow from the Ganges, large‐scale upstream withdrawal and massive sedimentation that shifts paths and forms char lands or temporary islands. This disrupts the migration and could lead fish of upstream to complete their life cycle within the upstream. However, during the rainy season water flows increase between upstream and downstream Padma and fish from downstream migrate to upstream leading to a diverse gene pool in the upstream Padma.

The results of AMOVA and F‐statistics demonstrate high genetic differentiation between Bangladeshi and Iraqi populations, while intraregional populations showed either lower but significant or nonsignificant genetic differentiation. The high level of inter‐regional differentiation likely resulted from reproductive isolation, driven by geographical and/or behavioral factors. The lack of gene flow has caused these populations to evolve independently, leading to distinct patterns of genetic drift, mutation, and selection pressures that contributed to significant differences in their genetic makeup. Similar genetic divergence was reported in other anadromous species, where physical barriers like dams and long geographic distances restricted gene flow (Bond et al. 2014; Ribolli et al. 2021; Sabatino et al. 2021). These findings align with those of Habib et al. (2022), who reported high population divergence and almost no migration of *T. ilisha* between the Arabian Gulf and the Bay of Bengal, and Behera et al. (2015), who similarly observed significant genetic structuring of *T. ilisha* populations in the Bay of Bengal and the Arabian Sea. In contrast to the high inter‐regional differentiation, AMOVA results revealed minimal genetic differentiation among Bangladeshi populations, indicated by low variation percentages (7.90%) (Table 3). This suggests weak population structuring among *T. ilisha* populations from different habitats, implying that these populations have maintained gene flow and resulting in higher levels of genetic homogeneity. Similarly, our F‐statistics analysis indicated lower differentiation among most Bangladeshi populations than higher inter‐regional population differentiation. The levels of genetic differentiation observed in the present study are consistent with the findings of Sarker et al. (2021), who reported low differentiation in Bangladeshi *T. ilisha* populations. This level of connectivity and differentiation points to a cohesive metapopulation structure in Bangladesh.

The most significant differentiation among Bangladeshi populations was observed between Hakaluki and Goalanda (F_st_ = 0.208, *p* = 0.00 ± 0.00). The Hakaluki and Goalanda populations belong to distinct river systems: Hakaluki is a part of a floodplain ecosystem within the Meghna River system, while Goalanda belongs to the Padma River system, a major distributary of the Indian Ganges River. This genetic differentiation suggests historical or reproductive isolation, resulting in partially distinct mitochondrial lineages between the two populations. The significant differentiation of the Teknaf population from other Bangladeshi populations also suggests that this population may be reproductively isolated. Based on these findings of historical or reproductive isolations, we hypothesize that *T. ilisha* females return to their natal habitats for spawning. The results further suggest the presence of more than one mitochondrial lineage of *T. ilisha* in Bangladeshi waters, which may share a relatively recent common ancestry and have not accumulated many mutations yet. Asaduzzaman, Wahab, et al. (2019); Asaduzzaman, Igarashi, et al. (2019) also suggested that *T. ilisha* exhibits natal homing behavior by returning to its natal river for reproduction.

Apart from this, the lowest genetic differentiation (F_st_ = 0.044, *p* = 0.0368 ± 0.0018) was observed between the Sundarbans and Hakaluki populations, despite being situated in distinct river systems (Pashur River in the Sundarbans is connected with the Padma River system and Hakaluki is in the Meghna River system) and contrasting locations (Table 4). Interestingly, some pairs of populations, such as Sundarbans and Chandpur or Sundarbans and Kuakata, did not show significant differentiation despite being part of distinct river systems. In contrast, populations within the same river system, such as Goalanda and Rajshahi or Hakaluki and Chandpur, showed some degree of differentiation. The presence of low or nonsignificant mitochondrial differentiation between some populations located in different river systems suggests historical connectivity and shared maternal ancestry. Conversely, the occurrence of differentiation among certain populations within the same river system may indicate localized reproductive structuring.

The population structure patterns observed here are in agreement with those reported by Asaduzzaman, Igarashi, et al. (2019) and Mohindra et al. (2019) who highlighted the impact of environmental influences and migratory behavior on the distinct population structure of *T. ilisha* within river systems. Although we observed substantial genetic differentiation among some Bangladeshi *T. ilisha* populations, we did not find significant differentiation between the two Iraqi populations, as indicated by both AMOVA and F‐statistics results. For Iraqi populations, the AMOVA showed that most of the genetic variation was within rather than among populations (Table 3). Apart from AMOVA, the F‐statistics supported these findings, showing no significant genetic differentiation between the two Iraqi populations (*p* = 0.162 ± 0.004). This lack of significant differentiation between Iraqi populations suggests a high degree of genetic homogeneity and potential shared ancestry within the region. These findings on Iraqi populations align with preliminary work by Abdullah et al. (2022), who reported genetic homogeneity across several locations along the Shatt al‐Arab River, including its estuarine zone.

This observed genetic uniformity in Iraq contrasted with the Bangladeshi populations, where some degree of genetic structuring was found. The Shatt al‐Arab River and the Arabian Gulf are facing serious ecological challenges resulting from a combination of human activities and natural environmental changes (Kadhim et al. 2020; Al‐Saad et al. 2022). The river has experienced a significant decline in water quality with escalating salinity levels, particularly from Basra to the river mouth (Abdullah et al. 2015). Long‐term reductions in river discharge impacted the Gulf's oceanographic conditions, affecting fisheries resources and overall marine biodiversity (Al‐Yamani et al. 2021). The low genetic diversity and lack of genetic differentiation likely resulted from small population size in the region, as indicated by the sharp decline in catch volume of this species (Mohamed 2022).

The results from our haplotype network analysis for the concatenated *COI* and *CYTB* sequences demonstrate significant inter‐ and intraregional historical gene flow patterns and demographic events, highlighting inter‐regional isolation and intraregional connectivity. The absence of shared haplotypes between Bangladeshi and Iraqi populations in the haplotype network indicates substantial genetic isolation and region‐specific evolution over time. The geographic and ecological separation between the Bay of Bengal and the Arabian Gulf likely acts as a barrier, limiting the movement and genetic exchange of *T. ilisha* populations (Bradburd et al. 2013). The inter‐regional isolation observed in the present study aligns with studies on other anadromous fish species such as Atlantic salmon, where significant genetic differentiation was observed due to natural geographic barriers limiting migration and gene flow (Dionne et al. 2008). However, within each region, there is evidence of shared haplotypes that suggest a historical maternal lineage relationship. The intricate branching pattern observed in the haplotype network of Bangladeshi populations may reflect a complex life history, including potential historical subdivision or varying migratory behaviors.

Significant Negative Fu's Fs values in Bangladeshi populations, except the Teknaf, support recent demographic expansions, as negative Fs values are indicative of excess low‐frequency mutations associated with population growth. This inference was further supported by nonsignificant SSD and Harpending's RI values in these populations, suggesting a good fit to the sudden expansion model. In contrast, in the Teknaf population, a positive (but nonsignificant) Tajima's D value, nonsignificant Negative Fu's Fs value, and multimodal mismatch distribution indicate no clear sign of recent demographic expansion and may reflect demographic stability. In Iraqi populations, nonsignificant Tajima's D and Fu's Fs values and more ragged mismatch distributions suggest demographic stability and the absence of recent population expansion. These findings suggest that several *T. ilisha* populations in Bangladesh might be benefiting from favorable environmental conditions that support demographic expansion and genetic diversification. The demographic pattern in Iraqi populations contrasts with that of Bangladeshi populations. The demographic expansion of the Bangladeshi populations occurred during the late Pleistocene and is attributed to a series of large glacial–interglacial cycles occurring at approximately 100,000‐year intervals (Imbrie et al. 1992). Climatic changes during this period led to variations in temperature and salinity, thereby influencing global ocean circulation patterns (Bond et al. 1997; Petit et al. 1999). During glacial periods, sea levels fell to approximately 120–140 m below present‐day levels, exposing extensive shallow water ecosystems. These changes significantly influenced marine population dynamics, leading to local extinction, range shifts, and recolonization (Hewitt 2000; Lambeck et al. 2002; Liu et al. 2006). During interglacial periods, sea levels rose, creating new habitats and altering ecosystems, thereby facilitating the expansion of fish populations. Based on our estimates of expansion times for *T. ilisha* populations (36,500–138,800 years ago), the most recent expansion likely occurred prior to the last glacial maximum (LGM: 18,000–26,500 years ago; Clark et al. 2009).

## 5 Conclusions

This study provides insights into the regional genetic diversity, population structure, and demographic history of *T. ilisha* . The findings have important implications for the sustainable management and conservation of *T. ilisha* stocks in both the Bay of Bengal and the Arabian Gulf regions. The significant genetic differentiation between distinct river systems suggests that Hilsa should not be managed as a single panmictic stock. Fisheries authorities should consider river‐specific conservation measures, such as site‐specific sanctuary periods that align with the spawning migrations of local populations. As the Teknaf estuarine population exhibited the highest genetic diversity, protecting this population is important for maintaining the adaptive potential of the species in the face of climate change. The genetic homogeneity and signs of historical bottlenecks in the Shatt al‐Arab indicate a population at risk. Management must prioritize restoring water quality to prevent further loss of genetic variation. Stricter harvest regulations and potentially “rest‐period” closures are needed to allow for population recovery and to prevent “inbreeding depression” or local extinction.

## Author Contributions

**Md. Nurul Alam:** conceptualization (equal), data curation (lead), formal analysis (lead), investigation (lead), methodology (equal), software (lead), writing – original draft (lead), writing – review and editing (equal). **Mohd Golam Quader Khan:** conceptualization (equal), funding acquisition (supporting), methodology (equal), resources (equal), supervision (supporting), writing – review and editing (equal). **Md. Bazlur Rahman Mollah:** conceptualization (equal), methodology (equal), resources (supporting), supervision (supporting), writing – review and editing (equal). **Md. Samsul Alam:** conceptualization (equal), methodology (equal), validation (equal), writing – review and editing (equal). **Sajid Saad Hasan:** conceptualization (equal), methodology (supporting), writing – review and editing (equal). **Md. Shahidul Islam:** conceptualization (lead), data curation (equal), formal analysis (equal), funding acquisition (lead), investigation (equal), methodology (equal), project administration (lead), resources (lead), software (equal), supervision (lead), validation (equal), writing – original draft (equal), writing – review and editing (equal).

## Funding

This work was supported by Bangladesh Academy of Sciences, 4th Phase BAS‐USDA Endowment Program (BAU FI 16).

## Conflicts of Interest

The authors declare no conflicts of interest.

## Supporting information


**Table S1:** Haplotype distribution of the concatenated mt DNA partial *COI* and *CYTB* gene in *T. ilisha* from different populations.

## Data Availability

The sequence data that support the findings of this study have been deposited in the NCBI GenBank database under accession numbers [COI: The GenBank accession numbers PP478830–PP479107; Cyt b: The GenBank accession numbers PP488948–PP489215]. These data are currently under embargo until the associated manuscript is published, after which they will be publicly available.
