# Supplementary material for: Genetic Diversity, Population Structure, and Demographic History of Anadromous Hilsa Shad (Tenualosa ilisha) Across Diverse Geographical Regions: A Comparative Study Between Bangladesh and Iraq
Source: Ecol Evol. 2026 Apr 22;16(4):e73524. doi: 10.1002/ece3.73524 (PMC13102496; doi:10.1002/ece3.73524)
Supplement: Supplementary file 1 — Table S1: Haplotype distribution of the concatenated mt DNA partial COI and CYTB gene in T. ilisha from different populations. [file ECE3-16-e73524-s001.docx]

**Appendix**

Table S1: Haplotype distribution of the concatenated mt DNA partial *COI* and *CYTB* gene in *T. ilisha* from different populations.

| Haplo-type | Chand-pur | Goa-landa | Hakaluki | Kuakata | Sundarbans | Rajshahi | Teknaf | Arabian Gulf | Shatt al-Arab | Total |
| --- | --- | --- | --- | --- | --- | --- | --- | --- | --- | --- |
| Hap_1 | 7 | 1 | 3 | 4 | 4 | 6 | -- | -- | -- | 25 |
| Hap_2 | 8 | 8 | 1 | -- | -- | 1 | 2 | -- | -- | 20 |
| Hap_3 | 1 | 1 | -- | -- | -- | 1 | -- | -- | -- | 3 |
| Hap_4 | 1 | -- | -- | -- | -- | -- | -- | -- | -- | 1 |
| Hap_5 | 1 | 4 | -- | -- | 2 | 1 | 2 | -- | -- | 10 |
| Hap_6 | 2 | 1 | 1 | 9 | 2 | 6 | 4 | -- | -- | 25 |
| Hap_7 | 2 | 3 | -- | -- | 2 | 1 | 1 | -- | -- | 9 |
| Hap_8 | 1 | 2 | -- | 1 | 1 | -- | -- | -- | -- | 5 |
| Hap_9 | 2 | -- | -- | -- | -- | 1 | -- | -- | -- | 3 |
| Hap_10 | 2 | 1 | 4 | -- | 3 | 1 | -- | -- | -- | 11 |
| Hap_11 | 1 | -- | -- | -- | -- | -- | -- | -- | -- | 1 |
| Hap_12 | 1 | -- | -- | -- | -- | -- | -- | -- | -- | 1 |
| Hap_13 | 1 | -- | -- | -- | -- | -- | -- | -- | -- | 1 |
| Hap_14 | -- | 1 | -- | -- | -- | -- | -- | -- | -- | 1 |
| Hap_15 | -- | 1 | -- | -- | -- | -- | -- | -- | -- | 1 |
| Hap_16 | -- | 1 | -- | -- | -- | -- | -- | -- | -- | 1 |
| Hap_17 | -- | 1 | -- | -- | 1 | -- | -- | -- | -- | 2 |
| Hap_18 | -- | 1 | -- | -- | -- | -- | -- | -- | -- | 1 |
| Hap_19 | -- | 1 | -- | -- | -- | -- | -- | -- | -- | 1 |
| Hap_20 | -- | 1 | -- | -- | -- | -- | -- | -- | -- | 1 |
| Hap_21 | -- | 1 | -- | -- | -- | -- | -- | -- | -- | 1 |
| Hap_22 | -- | -- | 1 | -- | -- | -- | -- | -- | -- | 1 |
| Hap_23 | -- | -- | 1 | -- | -- | -- | -- | -- | -- | 1 |
| Hap_24 | -- | -- | 1 | -- | -- | -- | -- | -- | -- | 1 |
| Hap_25 | -- | -- | 1 | -- | -- | -- | -- | -- | -- | 1 |
| Hap_26 | -- | -- | 2 | -- | -- | -- | -- | -- | -- | 2 |
| Hap_27 | -- | -- | 2 | -- | -- | 1 | -- | -- | -- | 3 |
| Hap_28 | -- | -- | 3 | 1 | -- | 1 | -- | -- | -- | 5 |
| Hap_29 | -- | -- | 1 | -- | -- | -- | -- | -- | -- | 1 |
| Hap_30 | -- | -- | 1 | -- | -- | -- | -- | -- | -- | 1 |
| Hap_31 | -- | -- | 1 | -- | -- | -- | -- | -- | -- | 1 |
| Hap_32 | -- | -- | 1 | -- | -- | -- | -- | -- | -- | 1 |
| Hap_33 | -- | -- | 2 | 2 | 1 | -- | -- | -- | -- | 5 |
| Hap_34 | -- | -- | 1 | -- | -- | -- | -- | -- | -- | 1 |
| Hap_35 | -- | -- | 1 | -- | -- | -- | -- | -- | -- | 1 |
| Hap_36 | -- | -- | 1 | -- | -- | -- | -- | -- | -- | 1 |
| Hap_37 | -- | -- | 1 | 2 | 1 | 1 | -- | -- | -- | 5 |
| Hap_38 | -- | -- | -- | 1 | -- | -- | -- | -- | -- | 1 |
| Hap_39 | -- | -- | -- | 1 | -- | -- | -- | -- | -- | 1 |
| Hap_40 | -- | -- | -- | 1 | -- | -- | -- | -- | -- | 1 |
| Hap_41 | -- | -- | -- | 2 | -- | -- | -- | -- | -- | 2 |
| Hap_42 | -- | -- | -- | 1 | -- | -- | -- | -- | -- | 1 |
| Hap_43 | -- | -- | -- | 1 | -- | -- | -- | -- | -- | 1 |
| Hap_44 | -- | -- | -- | 1 | -- | -- | -- | -- | -- | 1 |
| Hap_45 | -- | -- | -- | 1 | -- | -- | -- | -- | -- | 1 |
| Hap_46 | -- | -- | -- | 1 | -- | -- | -- | -- | -- | 1 |
| Hap_47 | -- | -- | -- | 1 | -- | -- | -- | -- | -- | 1 |
| Hap_48 | -- | -- | -- | -- | 1 | -- | -- | -- | -- | 1 |
| Hap_49 | -- | -- | -- | -- | 1 | -- | -- | -- | -- | 1 |
| Hap_50 | -- | -- | -- | -- | 1 | -- | -- | -- | -- | 1 |
| Hap_51 | -- | -- | -- | -- | 1 | -- | -- | -- | -- | 1 |
| Hap_52 | -- | -- | -- | -- | 1 | -- | -- | -- | -- | 1 |
| Hap_53 | -- | -- | -- | -- | 2 | -- | -- | -- | -- | 2 |
| Hap_54 | -- | -- | -- | -- | 1 | -- | -- | -- | -- | 1 |
| Hap_55 | -- | -- | -- | -- | 1 | -- | -- | -- | -- | 1 |
| Hap_56 | -- | -- | -- | -- | 1 | -- | -- | -- | -- | 1 |
| Hap_57 | -- | -- | -- | -- | 1 | -- | -- | -- | -- | 1 |
| Hap_58 | -- | -- | -- | -- | -- | 1 | -- | -- | -- | 1 |
| Hap_59 | -- | -- | -- | -- | -- | 1 | -- | -- | -- | 1 |
| Hap_60 | -- | -- | -- | -- | -- | 1 | -- | -- | -- | 1 |
| Hap_61 | -- | -- | -- | -- | -- | 1 | -- | -- | -- | 1 |
| Hap_62 | -- | -- | -- | -- | -- | 1 | -- | -- | -- | 1 |
| Hap_63 | -- | -- | -- | -- | -- | 1 | -- | -- | -- | 1 |
| Hap_64 | -- | -- | -- | -- | -- | 1 | -- | -- | -- | 1 |
| Hap_65 | -- | -- | -- | -- | -- | 1 | 1 | -- | -- | 2 |
| Hap_66 | -- | -- | -- | -- | -- | 1 | -- | -- | -- | 1 |
| Hap_67 | -- | -- | -- | -- | -- | -- | 2 | -- | -- | 2 |
| Hap_68 | -- | -- | -- | -- | -- | -- | 3 | -- | -- | 3 |
| Hap_69 | -- | -- | -- | -- | -- | -- | 1 | -- | -- | 1 |
| Hap_70 | -- | -- | -- | -- | -- | -- | 1 | -- | -- | 1 |
| Hap_71 | -- | -- | -- | -- | -- | -- | 3 | -- | -- | 3 |
| Hap_72 | -- | -- | -- | -- | -- | -- | 1 | -- | -- | 1 |
| Hap_73 | -- | -- | -- | -- | -- | -- | 1 | -- | -- | 1 |
| Hap_74 | -- | -- | -- | -- | -- | -- | 1 | -- | -- | 1 |
| Hap_75 | -- | -- | -- | -- | -- | -- | 1 | -- | -- | 1 |
| Hap_76 | -- | -- | -- | -- | -- | -- | 1 | -- | -- | 1 |
| Hap_77 | -- | -- | -- | -- | -- | -- | 1 | -- | -- | 1 |
| Hap_78 | -- | -- | -- | -- | -- | -- | 1 | -- | -- | 1 |
| Hap_79 | -- | -- | -- | -- | -- | -- | 1 | -- | -- | 1 |
| Hap_80 | -- | -- | -- | -- | -- | -- | 1 | -- | -- | 1 |
| Hap_81 | -- | -- | -- | -- | -- | -- | 1 | -- | -- | 1 |
| Hap_82 | -- | -- | -- | -- | -- | -- | 1 | -- | -- | 1 |
| Hap_83 | -- | -- | -- | -- | -- | -- | -- | 2 | -- | 2 |
| Hap_84 | -- | -- | -- | -- | -- | -- | -- | 9 | 16 | 25 |
| Hap_85 | -- | -- | -- | -- | -- | -- | -- | 15 | 11 | 26 |
| Hap_86 | -- | -- | -- | -- | -- | -- | -- | 2 | -- | 2 |
| Hap_87 | -- | -- | -- | -- | -- | -- | -- | 1 | -- | 1 |
| Hap_88 | -- | -- | -- | -- | -- | -- | -- | -- | 1 | 1 |
| Hap_89 | -- | -- | -- | -- | -- | -- | -- | -- | 1 | 1 |
| Hap_90 | -- | -- | -- | -- | -- | -- | -- | -- | 1 | 1 |
| Total | 30 | 29 | 30 | 30 | 28 | 30 | 31 | 29 | 30 | 267 |
